# Multimorbidity in bipolar disorder and undertreatment of cardiovascular disease: a cross sectional study

**DOI:** 10.1186/1741-7015-11-263

**Published:** 2013-12-23

**Authors:** Daniel J Smith, Daniel Martin, Gary McLean, Julie Langan, Bruce Guthrie, Stewart W Mercer

**Affiliations:** 1 Institute of Health and Wellbeing, University of Glasgow, Gartnavel Royal Hospital, 1055 Great Western Road, Glasgow G12 0XH, UK; 2 Institute of Health and Wellbeing, University of Glasgow, 1 Horselethill Road, Glasgow G12 9LX, UK; 3 Quality, Safety and Informatics Research Group, University of Dundee, Mackenzie Building, Kirsty Semple Way, Dundee DD2 4BF, UK

**Keywords:** Bipolar disorder, Coronary heart disease, Medication, Comorbidity

## Abstract

**Background:**

Individuals with serious mental disorders experience poor physical health, especially increased rates of cardiometabolic morbidity and premature morbidity. Recent evidence suggests that individuals with schizophrenia have numerous comorbid physical conditions that may be under-recorded and undertreated, but to date very few studies have explored this issue for bipolar disorder.

**Methods:**

We conducted a cross-sectional analysis of a dataset of 1,751,841 registered patients within 314 primary care practices in Scotland, UK. Bipolar disorder was identified using Read Codes recorded within electronic medical records. Data on 32 common chronic physical conditions were also assessed. Potential prescribing inequalities were evaluated by analysing prescribing data for coronary heart disease (CHD) and hypertension.

**Results:**

Compared to controls, individuals with bipolar disorder were significantly less likely to have no recorded physical conditions (OR 0.59, 95% CI 0.54 to 0.63) and significantly more likely to have one physical condition (OR 1.27, 95% CI 1.16 to 1.39), two physical conditions (OR 1.45, 95% CI 1.30 to 1.62) and three or more physical conditions (OR 1.44, 95% CI 1.30 to 1.64). People with bipolar disorder also had higher rates of thyroid disorders, chronic kidney disease, chronic pain, chronic obstructive airways disease and diabetes but, surprisingly, lower recorded rates of hypertension and atrial fibrillation. People with bipolar disorder and comorbid CHD or hypertension were significantly more likely to be prescribed no antihypertensive or cholesterol-lowering medications compared to controls, and bipolar individuals with CHD or hypertension were significantly less likely to be on two or more antihypertensive agents.

**Conclusions:**

Individuals with bipolar disorder are similar to individuals with schizophrenia in having a wide range of comorbid and multiple physical health conditions. They are also less likely than controls to have a primary-care record of cardiovascular conditions such as hypertension and atrial fibrillation. Those with a recorded diagnosis of CHD or hypertension were less likely to be treated with cardiovascular medications and were treated less intensively. This study highlights the high physical healthcare needs of people with bipolar disorder, and provides evidence for a systematic under-recognition and undertreatment of cardiovascular disease in this group.

## Background

Individuals with bipolar disorder have a standardised mortality rate (SMR) up to twice that of the general population [1-9]. Although suicide is a major cause of premature mortality in bipolar disorder, with a lifetime risk of around 8% [10], most of the excess mortality associated with bipolar disorder and similar serious major mental disorders is due to an increased prevalence of cardiovascular, metabolic and endocrine conditions and risky health behaviours [11,12]. Approximately 45% of individuals with bipolar disorder are known to smoke, compared with the population average of 20% [13]. It is also estimated that approximately 10% of people prescribed long-term antipsychotic medications will develop type II diabetes (at least twice the rate in the general population), and that 15% will develop hyperlipidaemia [14].

Despite higher rates of multiple medical comorbidities, there is also evidence that individuals with major mental illnesses receive less screening and fewer preventative interventions [15] and that they find it more difficult to implement lifestyle interventions aimed at modifying cardiometabolic risk factors [16]. A large population-based Danish study found that the recording of cardiovascular disease in individuals with bipolar disorder, schizophrenia and schizoaffective disorder was incomplete, and management insufficiently intensive [17]. A similar population-based cohort study in Sweden reported that despite elevated mortality rates (approximately double that of the general population) in bipolar disorder for cerebrovascular disease, coronary heart disease and acute myocardial infarction, hospital admission rates for cardiovascular disorder were only slightly increased compared to the general population. Taken together, these findings indicate a need for better recognition and treatment of physical health comorbidities in schizophrenia and bipolar disorder.

In a previous study we identified that individuals with schizophrenia in Scotland had significantly higher rates of multiple medical comorbidities but lower than expected rates of cardiovascular diseases recorded within primary care records [15]. Although it is reasonable to expect similar findings for bipolar disorder, to date there has been much less work carried out on the patterns and severity of multimorbidity in bipolar disorder. A fuller understanding of bipolar-specific patterns of comorbidity will help with the development of new integrated treatment approaches aimed at improving quality of life and reducing premature mortality.

In this study we aimed not only to examine the nature and extent of physical comorbidities in individuals with bipolar disorder within primary care but also to assess whether they experience inequitable prescribing for two key conditions: coronary heart disease and hypertension.

## Methods

We used a dataset from the Primary Care Clinical Informatics Unit at the University of Aberdeen, UK, consisting of all 1,751,841 registered patients who were alive and permanently registered with 314 general practices on 31 March 2007. This representative sample covers approximately one-third of the Scottish population. Data on the presence of 32 of the most common chronic physical health conditions were extracted (listed in Appendix 1). A more detailed explanation of how these conditions were selected and defined is available elsewhere [18].

People were identified as having bipolar disorder based on the recording at any point of any of the following primary care Read Codes (where % is noted this means ‘this code and any below it in the code hierarchy’): E110% Manic disorder, single episode/hypomanic psychosis; E111% Recurrent manic episodes; E114% Bipolar affective - now manic/manic depressive - now manic; E115% Manic-depression - now depressed; E116% Manic bipolar affective disorder; E117% Unspecified bipolar affect disorder; E11y; Other manic-depressive psychosis; E11y0 Unspecified manic-depressive psychosis; E11y1 Atypical manic disorder; Eu30% Manic episode; Eu31 Bipolar affective disorder/manic depressive illness/manic depressive psychosis; Eu323 Severe depressive + psychotic; and Eu333 Recurrent depression now severe + psychosis.

Deprivation status was measured using the Carstairs deprivation score which is widely used in healthcare research and is divided into quintiles to show differences between bipolar and non-bipolar patients, as well as for men and women with bipolar disorder [19]. Analysis was restricted to those aged at least 18 years old. The sample was divided into the following age groups for analysis: 18 to 24, 25 to 34, 35 to 44, 45 to 54, 55 to 64, 65 to 75, and 75 and over.

Differences between individuals with bipolar disorder and all other individuals, as well as differences between men and women with bipolar disorder, were calculated by age, deprivation status, number of physical conditions, cardiovascular risk factors (smoking, total serum cholesterol and blood pressure) and prescribing data. We used t tests to analyse differences between groups and one-way analysis of variance (ANOVA) for differences across age groups and deprivation quintiles. Age and gender specific rates were generated by the seven age groups outlined above. These age and gender standardised rates were then used to calculate odds ratio (ORs) and 95% confidence intervals (95% CIs) for those with bipolar disorder compared with those without bipolar disorder for the prevalence of all 32 physical conditions, as well as 0 physical disorders, 1 physical condition, 2 or more physical conditions and 3 or more physical conditions with results.

Standardised age and gender rates for risk factors and prescribing measures were also generated. For risk factors we compared people with coronary artery disease (CHD) and with hypertension with and without bipolar disorder in terms of whether they were current smokers, ever smokers, did not achieve target blood pressure (defined as systolic blood pressure (SBP) ≥140 mmHg and diastolic blood pressure (DBP) ≥90 mmHg if aged under 80 and SBP ≥150 mmHg and DBP ≥90 mmHg if aged 80 and over), or did not achieve total cholesterol ≤5 mmol/L (CHD analysis only). Differences in prescribing between those with and without bipolar disorder were also analysed by comparing the percentage of patients on aspirin/clopidogrel (for coronary heart disease only), a statin and antihypertensive drugs (defined as any angiotensin-converting enzyme inhibitor (ACEI) (all drugs in British National Formulary (BNF) issue 51, chapter 2.5.5.1), any angiotension II receptor blocker (ARB) (all drugs in BNF chapter 2.5.5.2), any beta blocker (all drugs in BNF chapter 2.4), any calcium-channel blocker (all drugs in BNF chapter 2.6.2), any alpha blocker (all drugs in BNF chapter 2.5.4), spironolactone (selected drug from BNF chapter 2.2.3), any other antihypertensive (all drugs in BNF chapter 2.5.1 or 2.5.2 or 2.5.3 or 2.5.4 not previously counted)). For all statistical analyses, a *P* value less than 0.05 was considered to be statistically significant. All analyses were performed in Stata version 11.1 (Stata Corp., College Station, TX, USA).

The UK National Health Service (NHS) National Research Ethics Service approved the anonymous use of these data for research purposes.

## Results and discussion

### Age, gender and deprivation status, bipolar versus non bipolar

We identified 2,582 people with a recorded bipolar read code (0.2% of the entire sample) and 1,421,796 without a bipolar read code (Table 1). Those with bipolar disorder were less likely to be male (39.5% of the bipolar group were male vs 49.1% of controls; *P* <0.001). Individuals with bipolar disorder tended to be older (mean age 54.5 years vs 47.9 years for controls; *P* <0.001), with bipolar patients less likely to be aged under 35 but more likely to be clustered in the 45 to 75 age range. Individuals with bipolar disorder were also less socially deprived (bipolar Carstairs score −0.31 vs. no bipolar −0.17; *P* <0.001) although differences between quintiles were marginal.

**Table 1:** Age, gender and deprivation status

**Variable**	**Bipolar, n (%)**	**Not bipolar, n (%)**	**Difference (95% CI) (*P *value)**
Total	2,582 (0.2)	1,421,796 (99.8)	
Gender (% male)	1,021 (39.5)	698,408 (49.1)	−9.6 (−7.6 to −11.5) (*P* <0.001)
Age, mean (SD)	54.5 (15.3)	47.9 (18.2)	6.5 (5.7 to 7.2) (*P* <0.001)
Deprivation, mean (SD)	−0.31 (3.3)	−0.17 (3.3)	0.14 (−0.8 to 0.1) (*P* = 0.03)
Age group:			
18 to 24	45 (1.7)	151,648 (10.7)	−9.0 (−7.8 to −10.1) (*P* <0.001)
25 to 34	217 (8.4)	229,179 (16.1)	−7.7 (−6.3 to −9.1) (*P* <0.001)
35 to 44	445 (17.2)	278,548 (19.6)	−2.4 (−0.1 to −3.9) (*P* = 0.01)
45 to 54	626 (24.2)	253,168 (17.8)	6.4 (5.0 to 7.9) (*P* <0.001)
55 to 64	547 (21.2)	218,786 (15.4)	5.8 (4.4 to 7.1) (*P* <0.001)
65 to 74	410 (15.9)	154,870 (10.9)	5.0 (3.8 to 6.2) (*P* <0.001)
75 and over	292 (11.3)	135,597 (9.5)	1.8 (0.1 to 2.9) (*P* <0.001)
Deprivation quintile:			
1 (least deprived)	513 (19.9)	271,516 (19.1)	0.8 (−0.2 to 1.4) (*P* = 0.32)
2	570 (22.1)	303,584 (21.4)	0.7 (−0.1 to 1.2) (*P* = 0.37)
3	581 (22.5)	321,666 (22.6)	−0.1 (−1.4 to 0.8) (*P* = 0.88)
4	514 (19.8)	270,870 (19.1)	0.8 (−0.2 to 1.6) (*P* = 0.23)
5 (most deprived)	404 (15.7)	254,160 (17.9)	2.2 (0.8 to 3.7) (*P* <0.001)

### Physical health comorbidity in people with bipolar disorder versus controls

Physical health comorbidities were very common for people with bipolar disorder, even after controlling for age and gender. Compared to controls, the bipolar group were significantly less likely to have 0 recorded physical conditions (OR 0.59, 95% CI 0.54 to 0.63; *P* <0.001) and significantly more likely to have 1 of the 32 most common physical conditions (OR 1.27, 95% CI 1.16 to 1.39; *P* <0.001), 2 physical comorbidities (OR 1.45, 95% CI 1.30 to 1.62; *P* <0.001) and 3 or more physical comorbidities (OR 1.44, 95% CI 1.30 to 1.64; *P* <0.001) (see Table 2).

**Table 2:** Prevalence and odds ratios for physical health comorbidity, standardised by age and gender

**Variable**	**Bipolar, n (%)**	**Not bipolar, n (%)**	**Odds ratio (95% CI) (*P *value)**
No physical condition	929 (36.0)	799,179 (56.2)	0.59 (0.54 to 0.63) (*P* <0.001)
One physical condition	662 (25.6)	292,651 (20.6)	1.27 (1.16 to 1.39) (*P* <0.001)
Two physical comorbidities	427 (16.5)	149,297 (10.5)	1.45 (1.30 to 1.62) (*P* <0.001)
Three or more physical comorbidities	564 (21.8)	180,669 (12.7)	1.44 (1.30 to 1.64) (*P* <0.001)
Individual conditions:			
Viral hepatitis	10 (0.4)	1,165 (0.1)	5.69 (3.22 to 10.01) (*P* <0.001)
Constipation	249 (9.4)	36,167 (2.5)	3.37 (2.93 to 3.88) (*P* <0.001)
Parkinson’s disease/Parkinsonism	19 (0.7)	2,722 (0.2)	3.05 (1.83 to 5.09) (*P* <0.001)
Chronic kidney disease	189 (7.3)	33,377 (2.4)	2.42 (2.04 to 2.86) (*P* <0.001)
Thyroid disorders	376 (14.6)	71,567 (5.0)	2.39 (2.11 to 2.70) (*P* <0.001)
Inflammatory bowel disease	27 (1.1)	9,724 (0.7)	1.99 (1.42 to 2.78) (*P* <0.001)
Epilepsy	47 (1.8)	12,337 (0.9)	1.98 (1.46 to 2.66) (*P* <0.001)
Pain	451 (17.5)	125,680 (8.8)	1.88 (1.69 to 2.09) (*P* <0.001)
Prostate disease	46 (1.8)	15,187 (1.1)	1.71 (1.28 to 2.29) (*P* <0.001)
Dyspepsia	237 (9.2)	78,967 (5.6)	1.63 (1.42 to 1.87) (*P* <0.001)
Blindness or low vision	30 (1.2)	8,348 (0.6)	1.58 (1.06 to 2.37) (*P* = 0.02)
Multiple sclerosis	16 (0.6)	3,831 (0.3)	1.58 (0.87 to 2.86) (*P* = 0.12)
Psoriasis or eczema	31 (1.2)	10,338 (0.7)	1.44 (0.98 to 2.10) (*P* = 0.06)
Chronic obstructive pulmonary disease	169 (6.6)	52,938 (3.7)	1.39 (1.17 to 1.65) (*P* <0.001)
Irritable bowel syndrome	148 (5.7)	51,989 (3.7)	1.37 (1.14 to 1.64) (*P* <0.001)
Diabetes	218 (8.4)	74,613 (5.3)	1.31 (1.13 to 1.53) (*P* <0.001)
Liver disease	6 (0.2)	2,608 (0.2)	1.26 (0.56 to 2.82) (*P* = 0.56)
Cancer	115 (4.5)	43,549 (3.1)	1.23 (1.00 to 1.54) (*P* = 0.04)
Hearing loss	134 (5.2)	54,600 (3.8)	1.18 (0.98 to 1.43) (*P* = 0.07)
Peripheral vascular disease	59 (2.3)	23,181 (1.6)	1.18 (0.93 to 1.56) (*P* = 0.22)
Asthma	179 (6.9)	83,809 (5.9)	1.16 (0.99 to 1.35) (*P* = 0.06)
Heart failure	47 (1.8)	18,852 (1.3)	1.11 (0.80 to 1.53) (*P* = 0.51)
Diverticular disease	89 (3.5)	33,724 (2.4)	1.11 (0.87 to 1.41) (*P* = 0.38)
Stroke and transient ischaemic attack	89 (3.5)	36,456 (2.6)	1.05 (0.84 to 1.34) (*P* = 0.63)
Inflammatory arthritis and related conditions	119 (4.6)	57,889 (4.1)	0.95 (0.78 to 1.17) (*P* = 0.67)
Coronary heart disease	170 (6.6)	81,297 (5.7)	0.94 (0.79 to 1.12) (*P* = 0.51)
Chronic sinusitis	17 (0.7)	9,148 (0.6)	0.90 (0.54 to 1.49) (*P* = 0.69)
Migraine	18 (0.7)	9,233 (0.7)	0.89 (0.54 to 1.49) (*P* = 0.67)
Hypertension	462 (17.9)	233,852 (16.5)	0.82 (0.73 to 0.92) (*P* <0.001)
Glaucoma	28 (1.1)	15,891 (1.2)	0.82 (0.55 to 1.24) (*P* = 0.36)
Atrial fibrillation	39 (1.4)	23,937 (1.7)	0.68 (0.45 to 0.94) (*P* = 0.02)
Bronchiectasis	5 (0.2)	2,809 (0.2)	0.78 (0.29 to 2.08) (*P* = 0.62)

**Figure 1:**
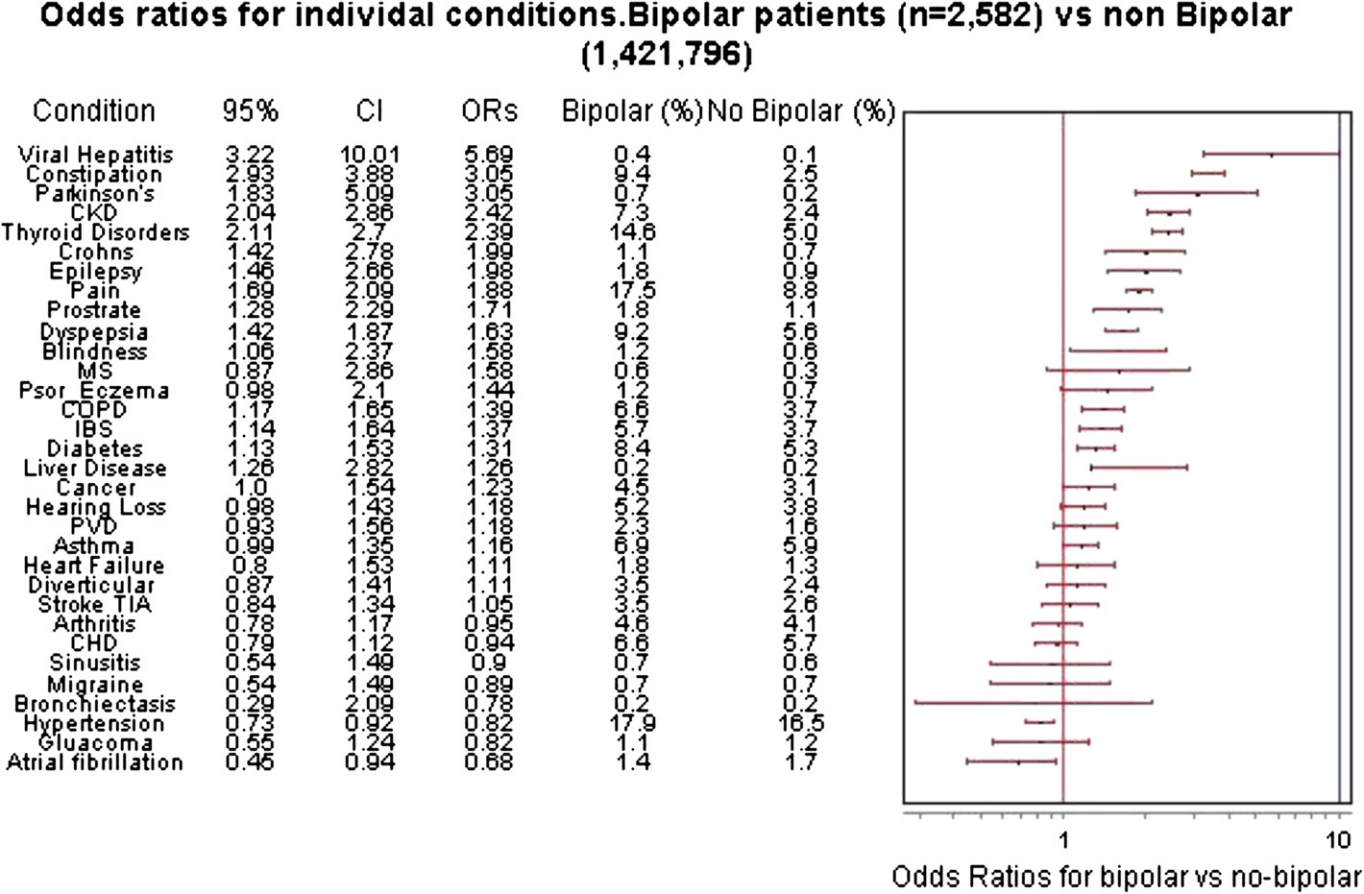
Odds ratios for individual conditions.

For each of the 32 individual physical conditions assessed, prevalence was significantly higher for individuals with bipolar disorder for 15 conditions, lower for 2 conditions and with no difference found for the remaining 15 conditions (Table 2). Prevalence was highest for bipolar versus non-bipolar for viral hepatitis (OR 5.69, 95% CI 3.22 to 10.01; *P* <0.001), constipation (OR 3.37, 95% CI 2.93 to 3.88; *P* <0.001) and Parkinson’s disease (OR 3.05, 95% CI 1.83 to 5.09; *P* <0.001) (see Figure 1).

People with bipolar disorder also had higher rates of several important chronic health conditions relevant to bipolar disorder for both unadjusted and adjusted results. This included chronic kidney disease (7.3% in bipolar disorder vs 2.4% in controls; OR 2.42 *P* <0.001), thyroid disorders (16.6% vs 5.0%; OR 2.39 *P* <0.001), chronic pain (17.5% vs 8.8%; OR 1.88 *P* <0.001), chronic obstructive pulmonary disease (COPD) (6.6% vs 3.7%; OR 1.39 *P* <0.001), and diabetes (8.4% vs 5.2%; OR 1.31 *P* <0.001).

The most commonly diagnosed condition for individuals with bipolar was hypertension (17.9%), but this was significantly lower than in the general population after controlling for age and gender, (OR 0.82, 95% CI 0.73 to 0.82; *P* <0.001). The only other physical condition in which the prevalence for bipolar patients was significantly lower following standardisation was atrial fibrillation (OR 0.68, 95% CI 0.45 to 0.94; *P* = 0.02).

### Cardiovascular risk factors and prescribing of cardiovascular medications

Table 3 shows differences in risk factors and prescribing levels for bipolar disorder and controls with coronary heart disease (CHD) and/or hypertension. Although the rate of ever smoking was very similar (67.6% for bipolar vs 63.3% for controls; *P* = 0.95), bipolar patients with CHD were more likely to be current smokers than controls (32% vs 20.6%; OR 1.55, 95% CI 1.11 to 2.17; *P* = 0.01). No difference was found for the achievement of cholesterol and blood pressure targets between individuals with bipolar disorder and controls. Bipolar patients with CHD were less likely to be on a statin (70.0% of bipolar vs 74.4% controls; OR 0.69, 95% CI 0.50 to 0.96; *P* = 0.02), more likely not to be on an antihypertensive (29.4% vs 15.8%; OR 2.08, 95% CI 1.49 to 2.91; *P* <0.001) and less likely to be on two or more antihypertensive medications (32.9% vs 52.7%; OR 0.46, 95% CI 0.33 to 0.63; *P* <0.001). There was no difference found for aspirin or clopidogrel prescribing.

**Table 3:** Differences in risk factors and prescribing between bipolar and controls for coronary heart disease (CHD) and hypertension patients standardised by age and gender

**Patients**	**Bipolar**	**Controls**	**Odds ratio (95% CI)**
CHD patients:	n = 170	n = 80,985	
Current smokers, %	32.3	20.6	1.55 (1.11 to 2.17) *P* = 0.01
Ever smokers, %	67.6	63.3	1.00 (0.73 to 1.37) *P* = 0.95
	n = 147	n = 73,508	
Total cholesterol >5, %	18.6	18.3	1.02 (0.73 to 1.37) *P* = 0.95
	n = 170	n = 80,863	
Blood pressure not controlled, %*	1.1	2.3	0.24 (0.03 to 1.74) *P* = 0.16
Prescribed drugs	n = 170	n = 81,297	
Aspirin or clopidogrel, %	69.3	73.6	0.81 (0.58 to 1.12) *P* = 0.20
Statin, %	70.0	74.9	0.69 (0.50 to 0.96) *P* = 0.02
No antihypertensive, %	29.4	15.8	2.08 (1.49 to 2.91) *P* <0.001
One antihypertensive, %	37.6	31.3	1.29 (0.94 to 1.76) *P* = 0.10
Two or more antihypertensives, %	32.9	52.7	0.46 (0.33 to 0.63) *P* <0.001
Hypertension patients:	n = 462	n = 232,986	
Current smokers, %	27.9	17.1	1.87 (1.53 to 2.39) *P* <0.001
Ever smokers, %	54.5	48.9	1.25 (1.04 to 1.50) *P* <0.001
	n = 462	n = 232.915	
Blood pressure not controlled, %*	6.5	6.6	0.94 (0.64 to 1.37) *P* = 0.77
Prescribed drugs	n = 462	n = 233,852	
Statin, %	36.7	41.5	0.82 (0.68 to 0.98) *P* = 0.04
No antihypertensive, %	21.8	13.9	1.70 (1.36 to 2.12) *P* <0.001
One antihypertensive, %	39.8	32.3	1.38 (1.15 to 1.67) *P* <0.001
Two or more antihypertensives, %	37.8	53.7	0.53 (0.44 to 0.68) *P* <0.001

Prescribing based on previous 84 days. Antihypertensives include any angiotensin-converting enzyme inhibitor (ACEI), angiotension II receptor blocker (ARB), beta blocker, calcium channel blocker, thiazide, alpha blocker, spironolactone or other antihypertensive.

*Blood pressure not controlled calculated as systolic blood pressure (SBP) ≥140 plus diastolic blood pressure (DBP) ≥90 if aged under 80, and SBP ≥150 plus DBP ≥90 if aged 80 and over.

Bipolar patients with hypertension were more likely to be current smokers (27.9% vs 17.1%; OR 1.87, 95% CI 1.53 to 2.39; *P* <0.001) and more likely to have ‘ever smoked’ (54.5% vs 48.9%; OR 1.25, 95% CI 1.04 to 1.50; *P* <0.001). They were less likely to be on a statin (36.7% vs 41.5%; OR 0.82, 95% CI 0.68 to 0.98; *P* = 0.04), more likely not to be on an antihypertensive agent (21.8% vs 13.9%; OR 1.70 95% CI 1.36 to 2.12; *P* <0.001) and less likely to be on two or more antihypertensive medications (37.8% vs 53.7%; OR 0.53, 95% CI 0.44 to 0.67; *P* <0.001).

### Gender differences within the bipolar group

We also assessed whether there might be differences between men and women with bipolar disorder in terms of the number of recorded physical health comorbidities, as well as age and deprivation status (Table 4). On average, women were older than men (55.8 to 52.4; *P* <0.001). Physical comorbidity was high in all bipolar patients, with two-thirds of both men and women having at least one recorded comorbid physical condition (Table 4). Women with bipolar disorder were significantly more likely to have three or more physical conditions than men (25.0% versus 17.0%; *P* <0.001).

**Table 4:** Differences between men and women with bipolar disorder

**Variable**	**Men, n (%)**	**Women, n (%)**	**Difference 95% CI (*P *value)**
Total	1,021 (39.5)	1,561 (60.5)	
Age, mean (SD)	52.4 (15.1)	55.8 (15.2)	−3.4 (2.2 to 4.5) (*P* <0.001)
Deprivation, mean (SD)	−0.40 (3.2)	−0.25 (3.4)	0.15 (−0.3 to 1.2) (*P* = 0.27)
Age group:			
18 to 24	20 (2.0)	25 (1.6)	0.4 (−0.3 to 0.7) (*P* = 0.49)
25 to 34	111 (10.9)	106 (6.8)	4.1 (1.8 to 6.2) (*P* <0.001)
35 to 44	195 (19.1)	250 (16.0)	3.1 (1.1 to 6.1) (*P* = 0.4)
45 to 54	253 (24.8)	373 (23.9)	0.9 (−0.4 to 2.4) (*P* = 0.60)
55 to 64	214 (21.0)	333 (21.3)	−0.3 (−0.2 to 3.5) (*P* = 0.82)
65 to 74	136 (13.3)	274 (17.6)	4.3 (1.3 to 7.1) (*P* <0.001)
75 and over	92 (9.0)	200 (12.8)	3.8 (1.3 to 6.9) (*P* <0.001)
Deprivation quintile			
1 (least deprived)	207 (20.3)	306 (19.6)	0.7 (−0.3 to 2.4) (*P* = 0.67)
2	228 (22.3)	342 (21.9)	0.4 (−0.3 to 2.8) (*P* = 0.80)
3	230 (22.5)	351 (22.5)	0.0 (−3.4 to 3.3) (*P* = 0.98)
4	213 (20.9)	301 (19.3)	1.6 (−0.4 to 2.3) (*P* = 0.33)
5 (most deprived)	143 (14.0)	261 (16.7)	−2.7 (−1.1 to 5.5) (*P* = 0.06)
Multimorbidity:			
No physical comorbidity	430 (42.1)	499 (32.0)	10.1 (6.3 to 13.9) (*P* <0.001)
One physical comorbidity	399 (25.8)	263 (25.6)	0.2 (−3.2 to −3.1) (*P* = 0.91)
Two physical comorbidities	154 (15.1)	273 (17.4)	−2.3 (−0.1 to 5.4) (*P* = 0.10)
Three physical comorbidities	174 (17.0)	390 (25.0)	−8.0 (4.6 to 11.1) (*P* <0.001)

## Discussion

### General findings

Our findings highlight that individuals with bipolar disorder have excess physical health comorbidities compared to the general population. Similar to our recent findings for schizophrenia [15], the majority of individuals with bipolar disorder (63.9%) had at least one chronic physical health comorbidity. Compared to patients without bipolar disorder, individuals with bipolar disorder were more likely to have a primary care record of both single and multiple physical health problems, even after taking into account age, gender and deprivation status. In general, our findings for bipolar disorder are similar to previous findings for schizophrenia, that is, for both conditions there appear to be high rates of multiple comorbidities and a systematic under-recording of cardiovascular conditions [15]. However, in the current paper we have extended this work by also identifying that individuals with bipolar disorder and a primary care record of coronary heart disease or hypertension tend to experience much less intensive prescribing for these conditions.

### Specific conditions

The three conditions with the highest prevalence within the bipolar group were viral hepatitis, constipation and Parkinson’s disease (which includes Parkinsonism). There were also high rates of diabetes, chronic pain and COPD. Perhaps at least partly as a consequence of known treatment side effects (for example, lithium therapy), thyroid disease and chronic kidney disease both also had higher prevalence, again after age/sex adjustment, compared to controls.

The relatively high rates of viral hepatitis, constipation and Parkinson’s disease may have a number of explanations. There are high rates of drug abuse in bipolar disorder [20] and within Scotland there is a national programme of testing for blood borne viruses in those diagnosed with drug dependence. This may explain the relatively high rates of viral hepatitis recorded in the bipolar group. Features of Parkinson’s disease can be a direct result of antipsychotic treatment. Although there is no clear mechanism for increased rates of constipation within bipolar disorder, psychological symptoms including those experienced as part of significant mood disorder are related to symptoms of irritable bowel syndrome including constipation [21,22]. Furthermore, constipation is an under-recognised side effect of antipsychotic medication that can significantly impact on quality of life, experienced by 20% to 30% of all patients prescribed such drugs [23].

### Recording of cardiovascular comorbidity

Rates of coronary heart disease, heart failure, stroke, and transient ischaemic attack (TIA) and peripheral vascular disease were not significantly elevated in the bipolar group. This is of note because rates of proatherosclerotic conditions including smoking and diabetes mellitus were found to be very significantly elevated in patients with bipolar disorder. Furthermore, individuals from the bipolar group displayed lower recorded rates of hypertension and atrial fibrillation compared to those without bipolar disorder. These findings might be unexpected given existing knowledge on physical comorbidity in bipolar disorder [11,12,15,16], but, as noted above, recent reports have highlighted that physical comorbidity in major mental illness is often underdiagnosed and undertreated [15-17,24].

There is strong evidence of under-recognition of cardiovascular disease in people with major mental illness. A recent Swedish national cohort study found that individuals with schizophrenia were more likely to die prematurely than the general population (15 years earlier for men and 12 years earlier for women) and the leading causes of death were cardiovascular disease and cancer. However, rates of recording of cardiovascular disease and cancer were only slightly increased in people with schizophrenia, even though these individuals had more healthcare system contacts, suggesting that cardiovascular disease and cancer are significantly underdiagnosed and/or under-recorded in this population [24]. Furthermore, using a similar approach to the one used in this study, we have identified an under-recording of cardiovascular illness in individuals with schizophrenia compared to expectations [15].

Our findings suggest a strong likelihood of under-recording of cardiovascular disease in bipolar disorder. There are a number of possible reasons for this health inequality. People with bipolar disorder may be less likely to seek help from their general practitioner (GP) with symptoms of cardiovascular disease because of low awareness of cardiovascular risk factors and associated symptoms [25]. This could be due to mental state abnormalities, social isolation and in some cases low levels of education. Cardiovascular risk estimation tools such as Framingham, ASSIGN and QRISK have not been validated in individuals with major mental illness [26] and may underestimate cardiovascular risk in bipolar patients, who are typically younger, smoke more and have higher blood pressure than the population traditionally used to generate the cardiovascular risk. This too may compound problems with under-recognition and undertreatment. People with bipolar disorder may also be prone to less regular attendance at clinics because of depressive and manic or hypomanic episodes [27].

### Prescribing of cardiovascular medications

A recent meta-analysis of prescribing data for patients with and without major mental illness found that individuals with a history of major mental illness had significantly lower prescription rates for cardiovascular medications [16]. Our findings indicate a similar prescribing disparity for both cholesterol lowering and antihypertensive medications. People with bipolar disorder and both coronary heart disease and hypertension had lower rates of statin prescription, as well as less intensive treatment with antihypertensive drugs. Although there was no evidence of different rates of poor blood pressure control and high cholesterol in a comparison between bipolar patients with CHD/hypertension comorbidity versus non-bipolar patients with CHD/hypertension, it is important to note that for people with CHD statins are recommended for all patients irrespective of pretreatment cholesterol level, so lack of prescription is indicative of a missed opportunity to deliver an evidence-based intervention. For people with hypertension, lower treatment intensity in the face of similar blood pressure or cholesterol control is more ambiguous. However, it is not possible to fully explore the relationship between treatment and outcome in cross-sectional data, particularly given the substantial premature cardiovascular mortality in bipolar disorder [1,12], consistent with people who were undertreated and with poor intermediate outcome control not surviving to be analysed in this kind of cross-sectional analysis. Our study cannot fully examine this issue, but highlights the importance of assessing treatment, intermediate outcome, and cardiovascular events and mortality within longitudinal research.

Although there are no universally accepted explanations for differences in prescribing for those with major mental illness, a number of possibilities exist. Mental health professionals may fail to appropriately diagnose physical health problems in their patients [28,29], they may carry out incomplete physical examinations [30] and a proportion may not feel confident with prescribing physical health (non-psychotropic) medications [16]. Despite this, most pharmacological treatments for cardiovascular illness take place in primary care. General practitioners who do not feel confident in managing complex and severe mental illness may be less likely to follow-up patients with major mental illness and comorbid physical health problems [31]. There are, however, conflicting reports within the literature that suggest that a higher frequency of healthcare visits in the context of mental illness may result in increases in guideline-recommended care for comorbid physical conditions [32]. Moreover, health care professionals working with individuals with major mental illness may be cautious when prescribing for this group due to concerns about suicide risk, as some cardiovascular medications, such as beta blockers, may be dangerous in overdose [16,33]. As noted above, it is also possible that concordance with healthcare provision, including attendance at GP and outpatient clinics, may be reduced in those with major mental illness [16]. Our results in relation to prescribing are potentially of concern because the adherence to cardiovascular medication in individuals with major mental illness may be even lower than the prescribing data suggests.

### Strengths and limitations

Strengths of this study include a very large sample (almost 1.8 million individuals) which is representative of the Scottish population and which has been selected from 314 primary care practices. It is worth noting that we have used routine data from primary care in this study rather conducting an epidemiological study. We believe that this large sample of ‘real world’ patients has some important advantages over an epidemiological study which, although more systematic with regards to diagnosis, will inevitably be smaller and almost certainly less representative because it will depend upon the referral of patients or volunteering patients.

Our use of Read Codes to identify both the index condition (bipolar disorder) and multiple medical comorbidities is potentially imperfect but represents the trade off between diagnostic accuracy and real-world representativeness and utility that is implicit in choosing between epidemiological studies and routine data studies. Clearly some estimates for some conditions may have been prone to bias. For example, as noted above, higher recorded rates of viral hepatitis and thyroid disease for bipolar patients may be related to more frequent blood monitoring within this group.

The rate of a recorded diagnosis of bipolar disorder of 0.2% is lower than might be expected, with most estimates of the prevalence of bipolar disorder being around 1% [34]. This likely reflects both underdiagnosis compared to epidemiological estimates, and under-recording reflecting the considerable debate and variation in the definitions and prevalence of the condition. However, this was not an epidemiological study but rather used routine clinical data on a very large number of participants. Our sample therefore represents the more severe end of the bipolar spectrum and we can be reasonably confident that most individuals recorded as having bipolar disorder did indeed have the condition.

It is possible that some patients with bipolar disorder are known to secondary care services but are not recorded within primary care and that a small additional proportion may not be in contact with either primary or secondary care. Further, these are routine data from 314 primary care practices and there may be some variability of diagnostic coding for major mental illnesses across these practices.

These data were cross-sectional, therefore no directionality or temporality can be attributed to the association between conditions, nor can any association confirm a causal relationship. It is possible that some of the comorbid medical conditions assessed may have predated or precipitated a diagnosis of bipolar disorder (and conversely that bipolar disorder was an antecedent for some of the medical conditions) but this is not something that can be adequately assessed using this cross-sectional dataset. All diagnoses were based on administrative data, and we did not have structured interviews to confirm their accuracy.

It should also be noted that an unknown (likely small) number of patients in our bipolar group had a read code of ‘severe/psychotic depression’. The prevalence of severe/psychotic depression in the general population is considerably lower than that of bipolar disorder and psychotic features are present in only a minority of cases of major depressive disorder [35]. There are also reports that indicate that severe or psychotic depression may be an early manifestation of bipolar disorder [36]. Unfortunately, due to limitations in the way these data were extracted it was not possible to separate out patients with psychotic depression from the bipolar group, although we expect that the proportion of patients in this sample with psychotic depression would have been very low, given that 11 out of the 13 Read Codes were for bipolar disorder diagnoses.

## Conclusions

In the UK, the NHS provides a free at the point of access healthcare system. Individuals should, in principle, have equal access to physical and psychiatric healthcare. The fundamental question that our study raises is whether this equal access translates into equal treatment and care for those with bipolar disorder [17].

Our results indicate that people with bipolar disorder have high rates of multiple comorbid physical health problems alongside underdiagnosis and/or under-recording of cardiovascular illnesses. Although rates of ever smoking were the same or similar in people with CHD and hypertension, people with these conditions and bipolar disorder were much more likely to be current smokers, which may indicate that health service smoking cessation services are insufficiently tailored to support this population. Furthermore, individuals with bipolar disorder with a recorded diagnosis of coronary heart disease or hypertension were less likely to be treated than those without mental illness and may be treated less intensively. This systematic under-recognition and undertreatment of cardiovascular disease may contribute to substantial premature mortality for individuals with a bipolar diagnosis in the UK.

It is well documented that physical and mental health problems interact to cause prolonged hospitalisation, treatment failure, poor quality of life and premature mortality [25,37,38]. The current separation between specialist physical and mental health services, and between primary and secondary care services in the UK and other countries, makes the coordinated care of the physical health of patients with bipolar disorder difficult. Several recent reports have highlighted that more integrated services are needed but how best to achieve this is unclear [39-41]. A thorough and detailed longitudinal analysis of possible reasons for this health inequality, alongside a multidisciplinary focus on improving physical health investigation, diagnosis, treatment and monitoring for those with bipolar disorder is needed.

## Appendix I

Table 5 contains details on how each of the 32 physical health conditions were defined from the primary healthcare records. In most cases these were based on Read codes and prescribing information.

**Table 5:** Definitions of 32 physical health conditions assessed

**Condition**	**Variable name**	**Variable definition**
Coronary heart disease	CHD	Read code ever recorded
Chronic kidney disease	CKD	Read code ever recorded
Asthma (active)	Asthma	Read code ever recorded AND any prescription in last year
Atrial fibrillation	Atrial fibrillation	Read code ever recorded
Epilepsy	Epilepsy	Read code ever recorded AND epilepsy prescription in last year
New cancer in the last 5 years	Cancer	Read code first recorded in last 5 years (Relevant Read Code recorded)
Thyrotoxicosis/thyroid disorders (includes hypothyroidism)	Thyroid Disorders	Read code ever recorded (Relevant Read Code recorded)
Diabetes	Diabetes	Read code ever recorded
Parkinson’s disease	Parkinson’s disease	Read code ever recorded (Relevant Read Code recorded)
Multiple sclerosis	Multiple sclerosis	Read code ever recorded (Relevant Read Code recorded)
Stroke or transient ischaemic attack	Stroke or TIA	Read code ever recorded (Relevant Read Code recorded)
Blindness and low vision	Blindness	Read code ever recorded (Relevant Read Code recorded)
Glaucoma	Glaucoma	Read code ever recorded (Relevant Read Code recorded)
Hearing loss	Hearing loss	Read code ever recorded (Relevant Read Code recorded)
Hypertension	Hypertension	Read code ever recorded (Relevant Read Code recorded)
Heart failure	Heart failure	Read code ever recorded
Peripheral vascular diseases	PVD	Read code ever recorded (Relevant Read Code recorded)
Chronic sinusitis	Sinusitis	Read code ever recorded (Relevant Read Code recorded)
Bronchitis, emphysema and other chronic obstructive pulmonary diseases	COPD	Read code ever recorded (Relevant Read Code recorded)
Bronchiectasis	Bronchiectasis	Read code ever recorded (Relevant Read Code recorded)
Crohn’s disease and ulcerative colitis	Inflammatory bowel disease	Read code ever recorded (Relevant Read Code recorded)
Diverticular disease of intestine	Diverticular disease	Read code ever recorded (Relevant Read Code recorded)
Rheumatoid arthritis, other inflammatory polyarthropathies and systematic connective tissue disorders	Inflammatory arthritis	Read code ever recorded (Relevant Read Code recorded)
Hyperplasia of prostate and prostate disorders	Prostate disease	Read code ever recorded (Relevant Read Code recorded)
Psoriasis or eczema	Psoriasis/eczema	Read code ever recorded (M11% and M12%) AND ≥4 prescriptions in last year (BNF 13.4, excluding hydrocortisone, and BNF 13.5)
Viral hepatitis	Viral hepatitis	Read code ever recorded (Relevant Read Code recorded)
Irritable bowel syndrome	Irritable bowel syndrome	Read code ever recorded (Relevant Read Code recorded) OR ≥4 antispasmodic prescription in last year (POM only, exclude kolanticon, alverine citrate and peppermint oil)
Cirrhosis/chronic liver disease/alcoholic liver disease	Chronic liver disease	Read code ever recorded (Relevant Read Code recorded)
Migraine	Migraine	≥4 antimigraine prescriptions in last year (BNF 040704%, POM only exclude migraleve)
Dyspepsia	Dyspepsia	≥4 prescriptions in last year BNF 0103% excluding antacids AND not ≥4 NSAIDs OR ≥4 aspirin/clopidogrel
Constipation	Constipation	≥4 prescriptions in last year, BNF 0106%
Pain	Pain	≥4 specified analgesic prescriptions in last year (opioids/>8 mg cocodamol/NSAIDs) OR ≥4 specified antiepileptics in the absence of an epilepsy Read code in last year (gabapentin, pregabalin and carbamazepine)

*BNF* British National Formulary, *NSAID* non-steroidal anti-inflammatory drug, *POM* prescription-only medicine.

## Competing interests

The authors declare that they have no competing interests.

## Authors’ contributions

GMcL carried out the analyses. DM and DJS and drafted the initial manuscript and all other authors contributed to subsequent drafts. All authors read and approved the final manuscript.

## Pre-publication history

The pre-publication history for this paper can be accessed here:

http://www.biomedcentral.com/1741-7015/11/263/prepub
